# Relative contribution of vitamin D deficiency to subclinical atherosclerosis in Indian context: Preliminary findings

**DOI:** 10.1097/MD.0000000000026916

**Published:** 2021-08-13

**Authors:** Srinivas Mantha, Sudha Lakshmi Tripuraneni, Lee A. Fleisher, Michael F. Roizen, Venkat Ramana Rao Mantha, Prasada Rao Dasari

**Affiliations:** a Division of Pain Medicine, Mantha Heart Clinic, Barkatpura, Hyderabad, India; b Division of Cardiology, Mantha Heart Clinic, Barkatpura, Hyderabad, India; c Department of Anesthesiology and Critical Care, Perelman School of Medicine, University of Pennsylvania, Philadelphia, PA; d Cleveland Clinic Lerner College of Medicine at Case Western Reserve University, Cleveland, OH; e Mantha Heart Clinic, Barkatpura, Hyderabad, India; f Indo-US Superspeciality Hospital, Ameerpet, Hyderabad, India.

**Keywords:** common carotid intima-media thickness, echo-tracking, India, preventive cardiology, subclinical atherosclerosis, ultrasound, Vitamin D3 deficiency

## Abstract

Asian Indians have a genetic predisposition to atherothrombotic risk. common carotid intima-media thickness (CCIMT) measured by ultrasound is a quantitative marker for atherosclerotic burden and a derived variable, that is, “CCIMT statistical Z-score (Z-score)” is useful for better quantification. The association between vitamin D deficiency and atherosclerosis is inconclusive. Since, vitamin D deficiency is highly prevalent in India, there is a need to study its relative contribution to subclinical atherosclerotic burden.

This prospective cross-sectional study (n = 117) in apparently healthy individuals aged 20 to 60 years sought to identify the determinants of CCIMT *Z* score with CCIMT measured by “echo-tracking” method. A multivariable linear regression analysis was done with CCIMT *Z* score as dependent variable and the following as independent variables: age, body mass index, waist-to-height ratio, total cholesterol to HDL ratio (TC-HDL ratio), serum vitamin D3 levels (ng/mL), sex, diabetes mellitus, current cigarette smoking status. A diagnostic prediction model was also developed with a threshold value of 1.96 for CCIMT *Z* score.

The mean (SD) for calendar age (y) was 40 (8). There were 26 (22.22%) individuals in sample with CCIMT *Z* score ≥1.96 (advanced stage) of whom 14 (23.33%) were <40 y (n = 60). The mean score was 1.28 (90th percentile) in the entire sample. Vitamin D3 deficiency with a mean (SD) blood level (ng/mL) of 14.3 (6.4) was noted and prevalence of deficiency was 81%. The final model was

CCIMT Z-score = 0.80 +  (0.841 × current smoking = 1) + (0.156 × TC-HDL ratio) – (0.0263 × vitamin D3 blood level in ng/mL).

The decreasing order of association is smoking, TC-HDL ratio, and vitamin D3. With the model, likelihood ratio (95% CIs) was better for positive test 3.5 (1.23–9.94) than that for a negative test 0.83 (0.66–1.02).

Internal validation with Bootstrap resampling revealed stability of baseline diagnostic variables.

There is substantial subclinical atherosclerotic burden in Indian setting with independent contribution by vitamin D deficiency. The model is valuable in “ruling-in” of the underlying advanced atherosclerosis. The study is limited by convenient sampling and lack of external validation of the model.

## 1 Introduction

Asian Indians have a genetic predisposition to atherothrombotic risk.^[1]^ Common carotid intima-media thickness (CCIMT) measured by ultrasound is a validated surrogate quantitative marker for atherosclerotic burden even at subclinical stage.^[2]^ Increased CCIMT is associated with future cardiovascular disease including stroke and coronary heart disease.^[3]^ As opposed to absolute thickness, a derived variable “CCIMT *Z*-score” is useful for better quantification since it is computed by comparing with age and sex matched population-based normal values.^[4]^ A CCIMT *Z* score of ≥1.96 equivalent to ≥97.5 percentile is defined as abnormal as it represents advanced stage that warrants attention and further evaluation.^[5]^ Earlier studies have used absolute thickness measured by off-line analysis of frozen images. In contrast, “echo-tracking” method that uses automated edge detection by real-time radiofrequency signal processing of ultrasound, is more sensitive and reliable.^[4]^

The association between vitamin D deficiency and atherosclerosis is contradictory and inconclusive.^[6]^ Emerging data has shown high prevalence of vitamin D deficiency among Indians despite the availability of abundant sunshine in large parts of India. Studies across India undertaken in diverse populations and geographical locations, both urban and rural, indicate a high prevalence of vitamin D deficiency in the range of 70% to 100%.^[7]^ There is a need for epidemiological survey to better address the relative contribution of vitamin D3 to subclinical atherosclerosis at finer levels in the Indian population.

Our primary aim was to identify the determinants of CCIMT *Z*-score and to predict it through a mathematical linear model.^[8]^ Such analysis helps to evaluate the relative contribution of vitamin D3, if any, among others to subclinical atherosclerosis, with thickness measured by echo-tracking method and CCIMT *Z*-score in the Indian population. The secondary aim was to develop a diagnostic prediction model of cardiovascular disease for young individuals in an otherwise relatively low risk asymptomatic individuals.^[9]^

## 2 Methods

### 2.1 Study design and analytic scheme (Supplemental digital content: Sections 1, 2 and 3)

The study was designed to identify determinants of CCIMT *Z*-score from among a set of predefined indicators in apparently healthy individuals. These indicators were selected a priori and defined in the protocol and analytic scheme was also prespecified. CCIMT was prospectively measured by ultrasound echo-tracking method

*Z*-score is estimated by the following formula^[4]^:

*Z*-score = (observed CCIMT − mean CCIMT)/SD CCIMT

Mean and standard deviation (SD) refer to mean and SD of CCIMT for that calendar age and sex.

Population reference standard values were those described by Engelen et al.^[4]^

A pilot series provided inputs for planning this study.^[10]^ (Supplemental digital content: Section 4) including sample size estimation (Supplemental digital content: Section 5). As a part of primary aim, multivariable linear regression analysis was done to model the relationship between CCIMT *Z* score as a dependent variable and a set of 8 predefined clinical and blood investigation findings as independent variables. The independent variables were age, body mass index (BMI), waist-to-height ratio (WHR), total cholesterol to HDL ratio (TC-HDL ratio), serum vitamin D3 levels (ng/mL), sex, diabetes mellitus, current cigarette smoking status. Current cigarette smoker was defined as the one who has smoked at least 100 cigarettes in the lifetime and who currently smokes cigarettes or has quit within the previous 12 months.^[11]^ The final linear model was developed from the identified determinants. The identification from initial analysis was based on statistical significance, standardized coefficients, evaluation of multicollinearity issue, and clinical relevance.

Development of diagnostic prediction model is intended to help clinical risk evaluation in the absence of ultrasound. Such potential application can be evaluated in terms of “ruling in” and “ruling out” of the underlying advanced atherosclerotic state. In this regard, a threshold value of 1.96 for both CCIMT Z-score obtained from direct ultrasound measurement and that estimated from the linear model to get a 2 × 2 table with cells depicting values for true positive, false positive, false negative, and true negative. Internal validation was done by Bootstrap resampling by repeating 10,000 times to assess the stability of baseline diagnostic variables.

### 2.2 Trail registration and subject enrollment

The study was approved by the Institute Ethics Committee at Indo-US Hospital, Ameerpet, Hyderabad, India. The study was registered prospectively in a clinical trial registry in India (http://ctri.nic.in) maintained by Indian Council of Medical Research. The registration number was CTRI/2019/02/017420, February 4, 2019 and study duration was expected to be 1 year (Supplemental digital content: Section 6). The Principal investigator was the first author (SM). Protocol summary may be viewed from the registry using “ccimt” in the key word search http://ctri.nic.in/Clinicaltrials/advancesearchmain.php.

The subject enrolment was prospectively performed between February 5, 2019 and December 31, 2019. The study was performed at two sites located in Hyderabad, south India, Indo-US Hospital, Ameerpet and Mantha Heart Clinic, Barkatpura (Supplemental digital content: Section 7). Inclusion criteria were subjects between 20 and 60 years of age who were healthy or with type 2 diabetes, hypertension or hypothyroidism with no end-organ damage and well controlled with oral medications, and BMI <40. Healthy state was also determined by the fact that they were living independently, were able to walk at least 4 blocks and could climb >2 floors of staircase (≥4 metabolic equivalents). Exclusion criteria were known cardiovascular disease or cerebrovascular disease, serum creatinine ≥2 mg/dL and those taking medications for dyslipidemia or history of treatment for vitamin D3 deficiency. Treatment for vitamin D3 deficiency was defined as oral intake of 60,000 IUs per week for at least 4 weeks during the past year. Patients with findings suggestive of myocardial ischemia on electrocardiogram (ECG) performed at the time of the study were excluded.

### 2.3 Protocol

After obtaining written informed consent, the subjects were screened for eligibility for the study. Blood for serum creatinine, lipid profile, and vitamin D3 were obtained as was an ECG. The subjects then underwent CCIMT measurement by ultrasound performed by one author (SM) experienced in the technique. The ECG was interpreted by one author (SLT) who is a cardiologist. The CCIMT ultrasound measurement was done before results of blood tests and ECG were available.

### 2.4 Study procedures and measurements

The CCIMT measurement was made by B-mode ultrasonography using 3 to 13 MHz linear probe. A new method called “echo-tracking” that uses automated edge detection by real-time radiofrequency signal processing of ultrasound was used. The region of interest is 1.5 cm starting from 1 cm of vertical reference line just proximal to carotid bulb. A table alongside the image gives measurements of last 6 cardiac cycles; each cardiac cycle is automatically detected by the arterial wall movement due to heart beats in the absence of ECG gating. Good quality measurement indicators are standard deviation <10 with a thick green overlay within the region of interest. The method is a patented technology of Esaote (Italy) and in the present series MyLab Gamma portable ultrasound machine was used.

Details of CCIMT measurement have been described in our previous paper^[10]^ and was performed with standard protocol.^[12]^ Briefly, the carotid artery is imaged in longitudinal view to visualize the “double line” sign representing the intima and media in the far wall of the common carotid artery. Specific to the “echo-tracking” method adopted in the present series, the operator obtains a real time feedback of measurement quality that helps to optimize ultrasound probe position.

### 2.5 Sample size estimation

The sample size required was calculated for 8 independent variables to be tested and no variables to be controlled in the multivariable linear regression analysis. Cohen medium “effect size” of 0.15 was used for a 2-sided alpha error of 0.05 and power of 0.8.^[13]^ As a result, the study required a minimal sample size of 111. An additional number of 11 (10%) was added to account for any exclusions that were possible after enrollment in the study. Hence, the study was planned for a total sample of 122 (Supplemental digital content: Sections 5).

### 2.6 Software for statistical analyses and reporting standards

PASS 16 Power Analysis and Sample Size Software (2018) (NCSS, LLC, Kaysville, UT, ncss.com/software/pass), was used for sample size estimation. NCSS 12 Statistical Software (2018) (NCSS, LLC. Kaysville, UT, ncss.com/software/ncss) for other standard analyses. An algorithm designed for R software (version 4.0.1, 2020, The R Foundation for Statistical Computing, Vienna, Austria) facilitated bootstrap resampling for validation of the diagnostic model. The presentation of this study is consistent with Strengthening the Reporting of Observational Studies in Epidemiology (STROBE) guidelines for the reporting of observational research.^[14]^ Specific to the secondary aim of study, it also adheres to several requirements for transparent reporting of a multivariable prediction model for individual prognosis or diagnosis (TRIPOD).^[15]^ In addition, as mandated for observational studies, detailed statistical analytic plan was described in the protocol and was available in the trail registration website and other public domains described in the data sharing statement.^[16]^

## 3 Results

### 3.1 Key findings

**Figure 1:**
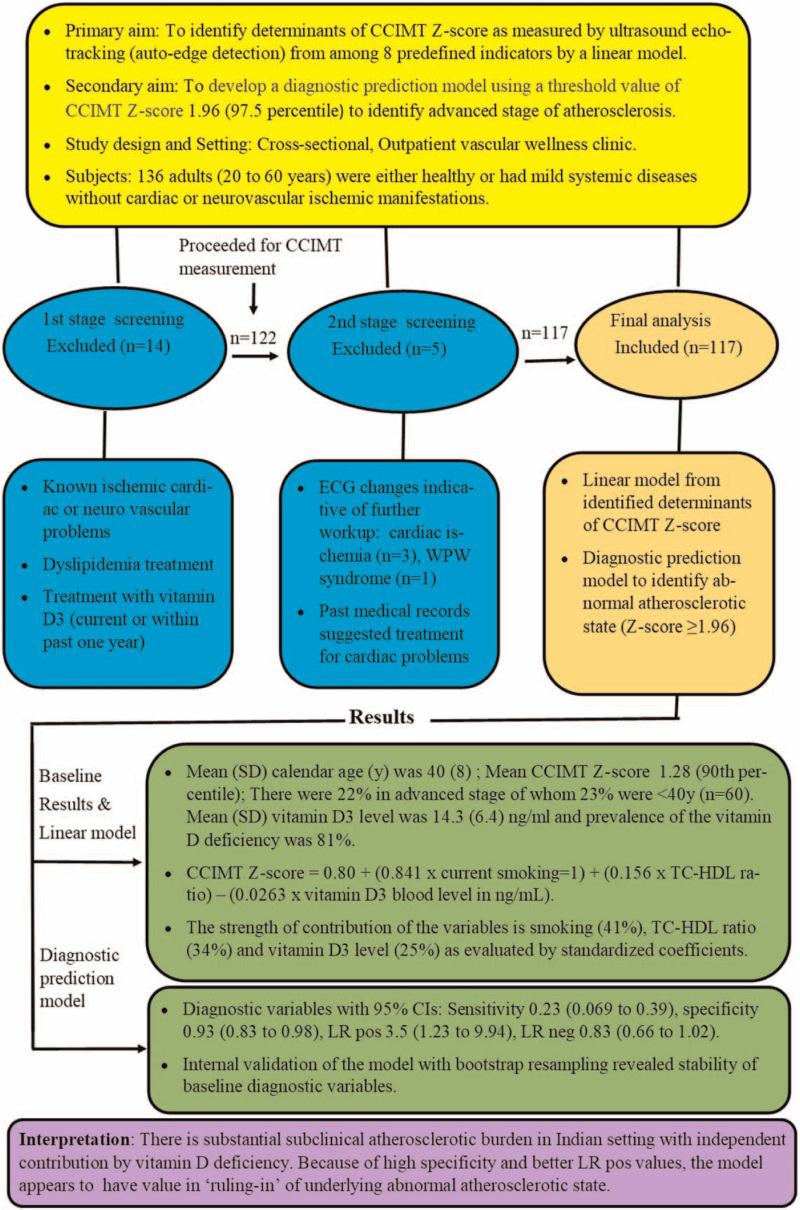
Summary overview: aims, design, setting, enrollment, analytic scheme, results, and interpretation. CCIMT = common carotid intima-media thickness, ECG = electrocardiogram.

**Figure 2:**
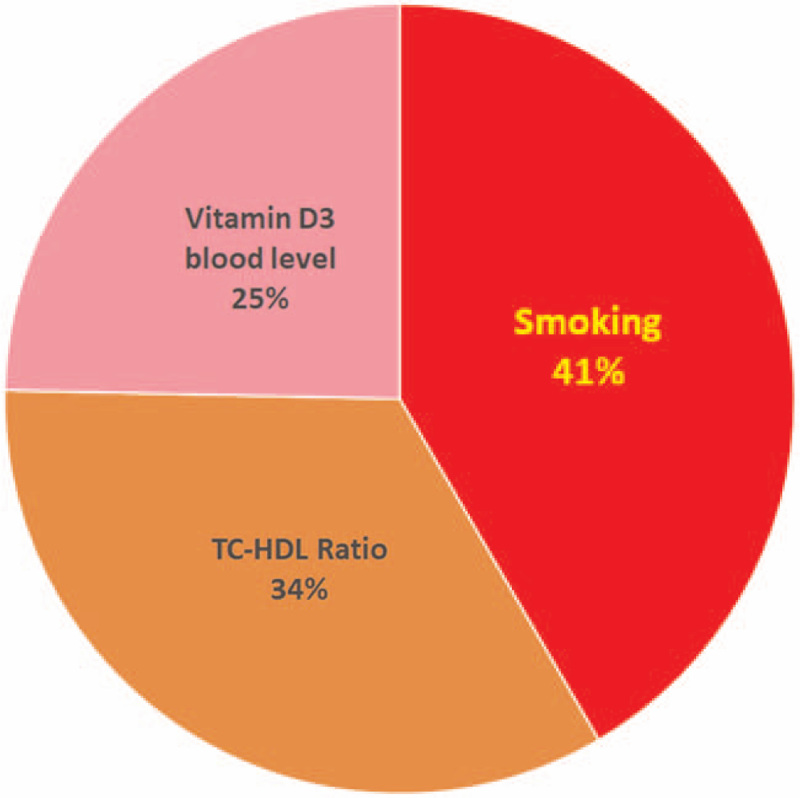
Relative contribution (weightage) of determinants of common carotid intima-media thickness (CCIMT) *Z* score. The weightage was determined by their standardized coefficients from multivariable linear regression model.

**Figure 3:**
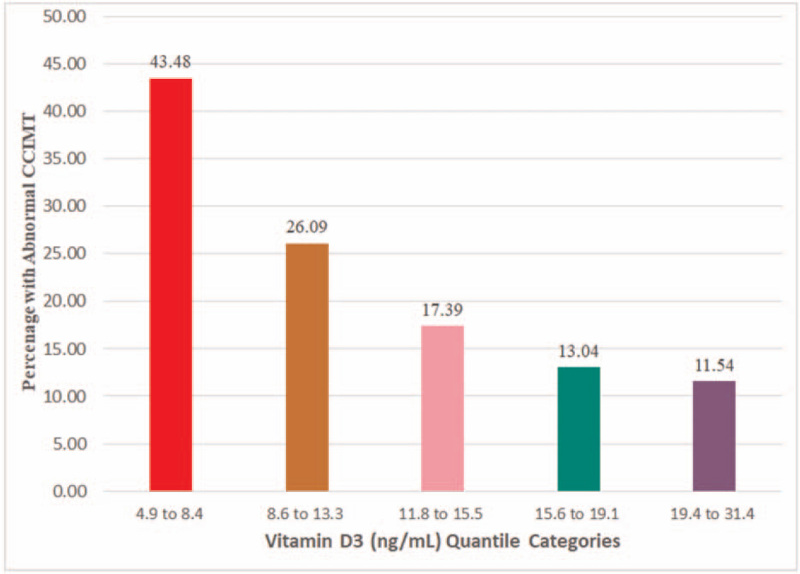
Percentage of individuals with abnormal (advanced stage) common carotid intima-media thickness (*Z* score ≥1.96) in each of the 5 quantile groups of serum vitamin D3.

A total of 117 subjects were studied, with a mean age (SD) of 40 years (8) years. Over all, there were 26 (22.22%) individuals with a CCIMT *Z* score of ≥1.96 of which 14 (23.33%) were <40 years (n = 60). Of the 8 predefined indicators tested, current smoking, TC-HDL ratio, and serum vitamin D3 were significantly associated with high CCIMT *Z* score through a linear model. Variance inflation factor analysis did not reveal existence of multicollinearity issue among the 8 tested indicators. The aims, design, setting, enrolment, and summary of the results are depicted in Fig. 1. Evaluation of standardized coefficients revealed weightage for smoking, TC-HDL ratio, and serum vitamin D3 levels in that decreasing rank order (Fig. 2). The proportion of individuals with abnormal CCIMT *Z* score progressively decreased with increasing vitamin D3 levels when evaluated with quantile groups (Fig. 3). Receiver operating characteristic curve (ROC) analysis with serum vitamin D3 levels and abnormal atherosclerotic state revealed a c-statistic of 0.68 (95% CI 0.55–0.80) with a cut-off value of 11.2 ng/mL for serum vitamin D3 level.

### 3.2 Baseline characteristics of the sample

Descriptive statistics for continuous data are shown in Table 1. The sample represents adults in an urban area in southern India. The mean (SD) calendar age was 40 (8) years. The mean CCIMT Z score of 1.28 corresponds to 90th percentile implying more atherosclerotic burden compared with western population standards. While BMI was normal there was a tendency for abdominal obesity with mean WHR of 0.58 (0.054). There was vitamin D3 deficiency in the sample with mean serum value being 14.3 (6.4) ng/mL (reference standards: sufficient [30–100], insufficient [20 to <30], deficient [<20], and prevalence of deficiency was 81%). Descriptive statistics of categorical variables were as follows (n = 117): male sex (57.30%), type 2 diabetes mellitus history (12%), hypertensive history (20.5%), current smoking status (12.8%), and hypothyroidism (9.4%). All smokers in our sample smoked even the day before the study. An incidental finding was that all subjects were right-handed and CCIMT was higher on the left side in 60% (71/117) (*P* = .02). The scan time (median and IQR) was 8 minutes (6–11), the minimum time was 2 minutes, and maximum time was 20 minutes.

**Table 1:** Baseline characteristics.

Variable	Mean (SD)	Median (IQR)	Minimum to maximum
Calendar age, yr	40 (8)	39 (34–46)	21–58
Body mass index	27 (4)	26 (24–29)	20–40
WHR	0.58 (0.054)	0.58 (0.55–0.62)	0.44–0.80
TC-HDL ratio	4.8 (1.5)	4.6 (3.6–5.7)	2.4–10.1
Vitamin D3, ng/mL	14.3 (6.4)	13.3 (9.4–18.9)	2.9–31.4
CCIMT Z-score^∗^	1.28 (0.95)	1.31 (0.58–1.86)	–0.87–4.06

### 3.3 Results of multivariable linear regression analysis for 8 predefined indicators

Initially, a multiple variable linear regression model was used to predict CCIMT *Z* score from the 8 predefined indicators. The model was statistically significant to predict CCIMT *Z* score: *F*-ratio (8, 108) = 4.101, *P* = .0003, coefficient of determination or adjusted *R*^2^ (adj.R^2^) = 0.176. Of the 8 indicators, 3 indicators (TC-HDL ratio, serum vitamin D3 level, and current smoking status), were significantly associated with CCIMT *Z* score. Variance inflation factor analysis did not reveal existence of multicollinearity issue among the 8 tested variables. (Supplemental digital content: Section 8). Hence, the model was analyzed using the 3 significant variables to obtain a final linear model to predict CCIMT *Z* score.

### 3.4 Results of multivariable linear regression analysis for 3 significant indicators

Results of the model with 3 indicators, that is, TC-HDL ratio, serum vitamin D3 level, and current smoking history status are presented in Table 2. This final model was statistically significant to predict CCIMT *Z* score from the identified 3 indicators: *F*-ratio (3, 113) = 9.775, *P* = .00001, coefficient of determination or adjusted *R*^2^ (adj.R^2^) = 0.185 with linear model as follows:

CCIMT *Z* score = 0.80 + (0.841 × smoking = 1) + (0.156 × TC-HDL ratio) – (0.0263 × vitamin D3 in ng/mL). (See Supplemental digital content: Section 9).

**Table 2:** Multivariable linear regression model for 3 identified indicators to predict common carotid intima-media thickness *Z* score.

Independent variable	Regression coefficient	Standardized regression coefficient	T statistic to test H0: β = 0	*P* value
Intercept	0.8	0	2.413	.0174
TC-HDL ratio	0.156	0.241	2.832	.0055
Serum Vitamin D3, ng/mL	–0.0263	–0.1759	–2.098	.0382
Current smoking	0.841	0.297	3.493	.0007

### 3.5 Results of diagnostic prediction model

**Figure 4:**
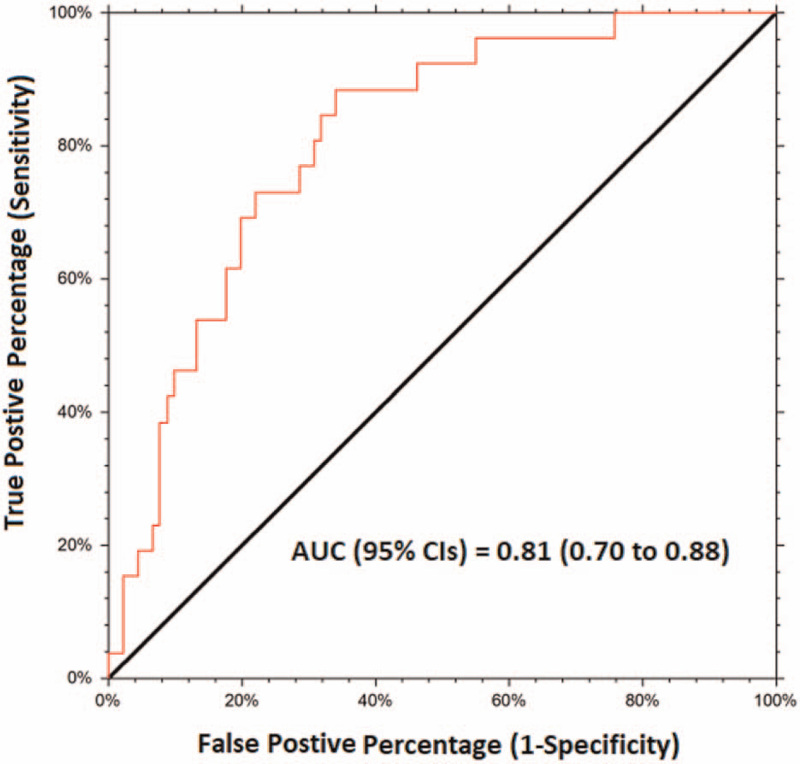
Receiver operating characteristic (ROC) curve for evaluation of model performance with regard to discrimination, that is, model estimated *Z* score to predict underlying advanced atherosclerotic state. The threshold for defining disease prevalence was common carotid intima-media thickness (CCIMT) *Z* score 1.96 (equivalent to 97.5 percentile) measured by ultrasound echo-tracking method (see text for details). The ROC curve shown is an “empirical curve” implying plot of true positives versus the false positives for all possible cut-off values. The area under the curve (AUC) was computed by empirical (nonparametric) method. The model had good discriminative power with area under receiver operating characteristic curve being 0.80 (95% CI 0.70–0.88).

The point estimate and 95% confidence intervals (CIs) for the clinically useful variables are presented in Fig. 1. With regard to performance, the model had good discriminative power with area under receiver operating characteristic curve being 0.80 (95% CI 0.70–0.88) (Fig. 4). Bootstrap resampling derived summary data for sensitivity, specificity, likelihood ratio for positive test, and likelihood ratio for a negative test are provided in Table 3.

**Table 3:** Results of bootstrap resampling for internal validation of the diagnostic prediction model.

	2.5thpercentile	25thpercentile	50th percentile	75th percentile	97.5th percentile
Sensitivity (%)	8	17	23	29	41
Specificity (%)	88	92	94	95	98
LR positive	1.08	2.54	3.5	5.25	15.17
LR negative	0.64	0.76	0.83	0.89	0.99

## 4 Discussion

The findings in the study indicate that current smoking, TC-HDL ratio, and vitamin D3 levels are important determinants of the CCIMT *Z* score even in apparently healthy individuals. In the sub-population without CVD and treatment (n = 14,609), both in men and Women CCIMT *Z*-scores were independently associated with systolic blood pressure, smoking, TC-HDL ratio, and BMI.^[4]^ In addition to the variables previously found to be associated with increased CCIMT Z score, WHR and serum vitamin D3 levels were also included. Smoking and high TC-HDL ratio (normal <4.5) are known risk factors for atherosclerosis. Indians have a genetic predisposition for central obesity that causes an increased risk factor for cardiovascular disease.^[1]^ Vitamin D deficiency also prevalent in Indians, is associated with cardiovascular disease and atherosclerosis.^[6]^ It was shown that treatment with vitamin D3 can significantly restore the damage to the cardiovascular system, while also reducing the risk of myocardial infarction.^[17]^ Dietary vitamin D intake appeared to be inversely associated with mortality from stroke.^[18]^ Our results include 3 indicators, TC-HDL ratio, serum vitamin D3, and current smoking status in the final linear model. These 3 had high standardized coefficients of 0.2 and above compared with calendar age which had a value of 0.14 and others which had a value <0.1. Variance inflation factor analysis did not reveal existence of multicollinearity issue among the 8 tested variables. In addition, age and sex are captured in the computation of CCIMT Z-score by comparing with calendar age and sex population standards. This method of selection of the determinants for the final model is more practical than other stepwise methods that involve forward selection and back elimination in multivariable analysis.

Risk prediction models offer an opportunity to identify a set of small but highly valid indicators in research settings. There are 2 types of risk prediction models, “diagnostic” and “prognostic.”^[9]^ Diagnostic models estimate the probability of having a pathological state or condition at a given time (cross-sectional), whereas prognostic models estimate the risk of developing a disease or outcome in the future (longitudinal). The models proceed from the stage of “development” to “validation” with the model performance assessed by various suitable statistical methods. In the present cross-sectional study, diagnostic prediction model is applicable. Such models help in evaluating the value of the diagnostic prediction in clinical practice by applying the principles of “ruling-in” and “ruling out” the underlying pathological state.^[19]^ Thus, they may enhance risk stratification in clinical practice. In these circumstances, the threshold value of CCIMT *Z* score used to define disease prevalence is important. While epidemiological studies use a threshold value of 75th percentile equivalent to *Z* score of 0.675 for defining abnormality to identify long-term risk,^[2]^ the present study used a threshold value of 97.5 percentile equivalent to 1.96 to capture more advanced subclinical atherosclerosis.

Finding of vitamin D3 deficiency in our sample is also not surprising but finding of inverse relationship of serum vitamin D3 levels and CCIMT *Z*-score is novel. Vitamin D deficiency is associated with incident cardiovascular disease and there is graded increase in risk across categories of deficiency for levels 10 to <15 ng/mL and for levels <10.^[20]^ In the Indian setting, severe vitamin D deficiency was associated with acute MI after adjusting for conventional risk factors.^[21]^ A recent study found that serum vitamin D3 level <20 ng/mL was not an independent risk factor for subclinical atherosclerosis in elderly north-east Asian population belonging I-Lan Longitudinal Aging Study group of Taiwan. The authors concluded that the association observed in the univariate analysis may be confounded by sex or comorbidities.^[22]^ Essential features and findings that could explain lack of independent association between vitamin D3 level and subclinical atherosclerosis are as follows: different ethnic and geographical group, less severe vitamin D deficiency (prevalence 33.6% and mean level 22.5 ng/mL), standard method of CCIMT measurement and measurement of absolute thickness. In contrast, in our study, the sample consisted of Asian Indians, there was more severe vitamin D deficiency (prevalence 81.2% and mean level 14.3 ng/mL), CCIMT measurement was by echo-tracking method which is more sensitive and reliable and derived variable of the thickness, CCIMT *Z* score that adjusts for age and sex. Our study specifically excluded those with vitamin D3 therapy currently or within the past 1 year.

Cardiovascular diseases have emerged as the leading cause of morbidity and mortality in India. Major modifiable risk factors include hypertension, dyslipidemia, diabetes mellitus, obesity, and lifestyle risk factors, such as smoking, inadequate physical activity, and less use of fresh vegetables and fruits. Present study used Engelen data for defining the population standards when computing *Z* score.^[4]^ The data collected by the Englen study was obtained primarily from European and some American research centers, with a relatively small minority of centers included in the study located outside of Europe (1 center was in Brazil and 1 in China out of 24 centers). A study provided age- and sex-specific distribution of CCIMT in Indian subjects free from cardiovascular disease.^[23]^ However, the study used standard method of measurement with off-line analysis software for CCIMT measurement as opposed to real-time echo-tracking with auto-edge detection method used in current study. In addition, data were presented in decade age-groups, for example, 30 to 39, 40 to 49, 50 to 59, and above 60 years. Lack of CCIMT data measured by echo-tracking in normal Indian population of either sex at all ages precluded use of such population data for computing the *Z* score. Hence, it is unclear from the current study, if this increased intimal thickness is due to a normal variation or a higher prevalence of cardiovascular disease in the studied sample. However, given the genetic predisposition to atherothrombotic risk^[1]^ and vitamin D deficiency observed in this study and other studies among Indians, the latter reason appears more likely. Further studies might throw more light on this matter. Data being preliminary in nature, it is premature to comment on handedness and predilection for higher CCIMT on the contralateral side. An earlier study has shown that the left side was thicker than the right between the ages of 35 and 65 years old.^[24]^

Findings in this study also have implications in perioperative medicine as both atherosclerosis and vitamin D3 deficiency are related to perioperative complications. Our pilot series proposed a research idea to use CCIMT data to quantify atherosclerotic burden as a part of preoperative cardiac risk stratification for noncardiac surgery.^[10]^ Such process could be helpful in shared decision making for perioperative care.^[25]^ The ischemia, in perioperative myocardial injuries, could be either due to supply-demand mismatch or thrombosis.^[26]^ Obviously, underlying coronary atherosclerosis can potentiate the problem. It is pertinent to note that there is a higher incidence of myocardial injury after noncardiac surgery in Indian setting compared with western data perhaps related more atherosclerotic burden.^[27]^ Hence, when applying the traditional cardiac risk stratification tools for noncardiac surgery^[28]^ in the Indian setting, one could use higher grade of systemic illness for individuals with CCIMT *Z* score ≥1.96. Alternatively, the indications for preoperative N-Terminal Pro-B-Type Natriuretic Peptide and periopreative troponin monitoring^[29]^ could be considered in those with *Z* score ≥1.96. Of course, larger outcome studies in the Indian population are warranted to make such recommendations. A recent study found a significant association between vitamin D deficiency and a composite of infectious complications and decreased kidney function although there was no relation to postoperative 30-day cardiac outcomes.^[30]^ Concepts of “prehabilitation”^[31]^ and “preoperative optimization”^[32]^ currently are gaining momentum with primary aim of perioperative risk attenuation given time of few weeks for proper evaluation and further necessary actions. Distinct advantage of ultrasound measurement of CCIMT is direct visualization of intima and affords planning strategies to attenuate the risk.^[10]^

Sensitivity analysis with Bootstrap resampling indicate stability of baseline diagnostic variables (point estimates and CIs) as they correspond to resampling distribution data. Better likelihood ratio for a positive test than that for a negative test implies that the model is useful in “ruling-in” than “ruling out” of underlying high atherosclerotic burden. Thus, in the absence of ultrasound, model could be used for initial risk assessment with the understanding that there are less chances of error due to false positives.

Limitations of the study primarily relate to sample and validation of the model. The sample (n = 117) represents a convenient sample of an urban population at the places of work of the first and second authors. But use of a sensitive and reliable method of CCIMT measurement coupled with controlling interoperator variability with all measurements made by a single experienced person strengthen the findings of this study. Ideally, models require external validation with independent sample that is lacking in this study. However, internal validation with Bootstrapping used in this study is believed more reliable than split sample method and other methods of internal validation.^[9]^ Emerging data suggest the role of nonalcoholic fatty liver disease (NAFLD) as an important mediator of subclinical atherosclerosis as well as atherosclerotic cardiovascular events.^[33]^ The role of NAFLD in vitamin D deficiency was also speculated.^[34]^ Studying those aspects of NAFLD related to subclinical atherosclerosis and vitamin D deficiency was beyond the scope of this study and may be areas for future research, hopefully stimulated by this report.

In conclusion, there is substantial subclinical atherosclerotic burden in Indian setting with independent contribution by vitamin D deficiency. A CCIMT *Z*-score of ≥1.96 (≥97.5 percentile) that represents advanced stage and warrants attention and further evaluation was found in 22% of our sample (n = 117) in apparently healthy individuals. Current smoking, TC-HDL ratio and vitamin D3 levels were significantly associated with CCIMT *Z* score and related with a linear model. The decreasing order of association is smoking, TC-HDL ratio and vitamin D3 as evaluated by standardized coefficients. In the absence of ultrasound, the model could be used for initial risk assessment with the understanding that there are less chances of error due to false positives. Further research is required for external validation of this model. Cohort studies may also be required to examine whether vitamin D3 therapy reduces carotid intimal thickness.

## Acknowledgments

Authors acknowledge the help from ClinArion Research Pvt. Ltd, Hyderabad, India, a site management organization (SMO), for handling the paperwork related to the study. Mr. Suresh Babu Allu, Ms. Anjali Ch, and Ms. Saleha Sultana of the organization also helped in coordinating the study at Indo-US Hospital, Ameerpet, Hyderabad, India. Authors thank Ms. G. Mamatha and K. Venkatesh Babu for help in coordination at Mantha Heart Clinic.

## Author contributions

Srinivas Mantha, MD: This author conceived the research idea, performed the ultrasound examination and measurement of IMT in all the subjects. He collected the data, performed the statistical data analyses, and wrote the initial draft of the manuscript.

Sudha Lakshmi Tripuraneni, MD: This author screened the subjects at Mantha Heart Clinic, Hyderabad, India and interpreted the ECGs of all the subjects. She helped revise the manuscript.

Lee A. Fleisher, MD: This author helped to improve the analysis and presentation of the manuscript.

Michael F Roizen, MD: This author had provided useful inputs to revise the manuscript substantially.

Venkat Ramana Rao Mantha, FFARCSI: This author helped in coordinating the cases at Indo-US Hospital, Hyderabad, India during June-July 2019 and also provided useful inputs to revise the manuscript.

Prasada Rao Dasari, MS, MCh: This author helped in coordinating the cases at Indo-US Hospital, Hyderabad, India.

**Data curation:** Srinivas Mantha.

**Formal analysis:** Srinivas Mantha.

**Investigation:** Srinivas Mantha.

**Methodology:** Srinivas Mantha, Sudha Lakshmi Tripuraneni.

**Project administration:** Srinivas Mantha, Sudha Lakshmi Tripuraneni, Venkat Ramana Mantha, Prasada Rao Dasari.

**Resources:** Srinivas Mantha, Sudha Lakshmi Tripuraneni.

**Software:** Srinivas Mantha.

**Supervision:** Srinivas Mantha.

**Validation:** Srinivas Mantha.

**Visualization:** Lee A Fleisher, Michael F. Roizen.

**Writing – original draft:** Srinivas Mantha.

**Writing – review & editing:** Sudha Lakshmi Tripuraneni, Lee A Fleisher, Michael F. Roizen, Venkat Ramana Mantha, Prasada Rao Dasari.

## Supplementary Material

Supplemental Digital Content
