# Supplementary material for: Relative contribution of vitamin D deficiency to subclinical atherosclerosis in Indian context: Preliminary findings
Source: Medicine (Baltimore). 2021 Aug 13;100(32):e26916. doi: 10.1097/MD.0000000000026916 (PMC8360406; doi:10.1097/MD.0000000000026916)
Supplement: Supplemental Digital Content [file medi-100-e26916-s001.docx]

**Supplemental digital content**

Relative contribution of vitamin D deficiency to subclinical atherosclerosis in Indian context:  Preliminary findings. Mantha S et al.

| **Section** | **Item** |
| --- | --- |
| 1 | Background |
| 2 | Echo-tracking method of CCIMT measurement |
| 3 | CCIMT Z-score |
| 4 | Linear regression analysis of 22 cases in the Pilot Series |
| 5 | Sample size estimation for the current study |
| 6 | Protocol, Ethics Committee and Trial Registration |
| 7 | Subject Enrolment and study sites |
| 8 | Initial Multivariable Linear Regression Analysis with 8 Predefined Indicators |
| 9 | Final multiple linear regression analysis with 3 selected indicators |
| 10 | Appendix: Example of CCIMT measurement by echo-tracking and generated data |

CCIMT = Carotid Intima-Media Thickness

**Data sharing**

Datasets generated and analyzed along with raw data and other details of the study are available at the website

[www.suhitam.com/vascularage](http://www.suhitam.com/vascularage)

Visitors of the website can login using ‘visitor’ for both user id and password to view the data. An alternate website [www.manthaclinics.com/vascularage](http://www.manthaclinics.com/vascularage) provides the same information.

1. **Background**

Common Carotid Intima-media Thickness (CCIMT) measured by ultrasound is a validated surrogate quantitative marker for atherosclerotic burden even at subclinical stage and usually measured in micrometers (µm).^1^ Traditionally, the CCIMT is measured by off-line analysis from frozen images. To circumvent the problems related to variability of measurement of CCIMT, a new method called ‘echo-tracking’ is now available. The method uses automated edge detection by radiofrequency real-time signal processing of ultrasound. The methodology ensures accuracy and reliability. Absolute CCIMT values are difficult to interpret in a given individual as CCIMT increases progressively with age at different rates in men and women. Hence, a derived variable, 'CCIMT Z-score' is useful for better quantification since it is computed by comparing with age and gender matched population-based normal values.^2^

The present study was designed to identify determinants of CCIMT Z-score (depended variable) with the thickness measured by ‘echo-tracking’ method in apparently healthy individuals. The 8 indicator variables (independent) were selected *a priori* and defined in the protocol and analytic scheme was also specified. A separate pilot series provided inputs for planning this study. including selection of indicator variables and sample size estimation.^3^ Usually, epidemiological studies use a threshold value of 75^th^ percentile (Z-score value of 0.675) for identifying individuals at long-term risk.^1^ However, the present study used a threshold value of 97.5 (Z-score value of 1.96) to capture more advanced subclinical atherosclerosis that warrants attention and further evaluation.^4^ The study was designed to identify determinants of CCIMT Z-score from among a set of predefined indicators in apparently healthy individuals.

CCIMT Measurement by Echo-tracking

The CCIMT measurement was made by B-mode ultrasonography using 3-13 MHz linear probe. In the echo-tracking method, the region of interest is 1.5 cm starting from 1 cm of vertical reference line just proximal to carotid bulb. A table alongside of image gives measurements of last 6 cardiac cycles; Each cardiac cycle is automatically detected by the arterial wall movement due to heart beats. Absent an ECG; good quality measurement indicators are SD less than 10 with a thick green overlay within the region of interest. The method is a patented technology of Esaote (Italy) and in the present series, MyLab Gamma portable ultrasound machine was used. Further technical details of CCIMT measurement by echo-tracking are available in our previous pilot series.^3^

1. **Concept of CCIMT Z-score**

CCIMT is usually measured in µm (micrometers). In both men and women, the IMT increases progressively with age, e.g. 5.2 µm/year in men and 5.0 µm/year in women and can be estimated as follows ^2^

In men,

mean CCIMT (µm) = 323.5 + 5.201 x age

standard deviation (SD) CCIMT (µm) = 57.24 + 0.9027 x age

In women,

mean CCIMT (µm) = 321.7 + 4.971 x age

SD CCIMT (µm) = 54.50 + 0.8256 x age

Since the CCIMT values increase progressively with age, absolute values are difficult to interpret in a given individual. Hence derived variable from CCIMT are commonly used in clinical practice to quantify the extent of atherosclerosis in the entire vascular tree. They are z-score (z) and vascular age

*
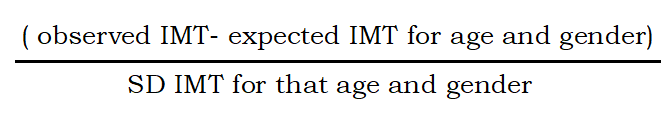
CCIMT Z-score*

[
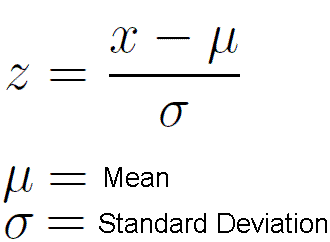
](https://www.google.co.in/url?sa=i&rct=j&q=&esrc=s&source=imgres&cd=&cad=rja&uact=8&ved=2ahUKEwjIseu3x57fAhXMpY8KHaSvBmEQjRx6BAgBEAU&url=https://www.thoughtco.com/z-score-formula-3126281&psig=AOvVaw0ulQ_YQ15W8qynWsVAaeQY&ust=1544850691553396)

- Man aged 34 years having

As typical in any standard normal distribution, a CCIMT Z score of zero (50^th^ percentile) represents thickness same as expected for that age and gender, a score of 0.675 (75^th^ percentile) represents more thickness and a score of 1.96 (97.5 percentile) represents most thickness deviation than expected.

1. **Linear Model from analysis of 22 cases in the Pilot Series**

There were four cases in this low-risk group (n=22) who had z score ≥1.96. A multiple variable linear regression model was used to predict CCIMT Z score from the following 5 predefined indicators: 4 continuous variables, i.e. BMI, WHR, TC-HDL ratio, serum vitamin D3. The model was statistically significant to predict CCIMT Z score: F-ratio (4, 17) = 3.295, *p* =0.0358, coefficient of determination or adjusted R^2^ (adj.R^2^)=0.3042. Of the 4 indicators, TC-HDL ratio was significantly associated to predict the CCIMT Z score with a P value of 0.0134. Although, not significant (p=0.19), next close variable based on standardized coefficient was vitamin D3.

Formal report from NCSS software is given below:

**Multiple Regression Report** **for Pilot Series (n=22)**

Dataset C:\...\2018_CCIMT_DemoStudyResults\20181215_ZscoreMVA.NCSS

Dependent IMT_Zscore

**Run Summary Report ─────────────────────────────────────────────────────────**

**Item Value Rows Value**

Dependent Variable IMT_Zscore Rows Processed 22

Number of Independent Variables 4 Rows Filtered Out 0

R² 0.4367 Rows with X's Missing 0

Adjusted R² 0.3042 Rows with Y Missing 0

Mean Square Error 0.453643 Rows used in Estimation 22

Completion Status Normal Completion

**Descriptive Statistics ─────────────────────────────────────────────────────────**

**Standard**

**Variable Count Mean Deviation Minimum Maximum**

TC_HDL_Ratio 22 4.790909 1.419225 3 8

BMI 22 28.18182 5.297226 19 37

Waist_Ht_Ratio 22 0.6027273 0.08674987 0.43 0.71

VitD3 22 17.94909 9.207312 7.5 50

IMT_Zscore 22 1.513864 0.8074464 0.638 4.47

**Regression Coefficients T-Tests ──────────────────────────────────────────────────**

**Regression Standard Standard- T-Statistic Reject**

**Independent Coefficient Error ized to Test Prob H0 at**

**Variable b(i) Sb(i) Coefficient H0: β(i)=0 Level 5%?**

Intercept 0.6926995 1.591695 0.0000 0.435 0.6689 No

TC_HDL_Ratio 0.3124295 0.1131689 0.5491 2.761 0.0134 Yes

BMI -0.02892174 0.05075173 -0.1897 -0.570 0.5762 No

Waist_Ht_Ratio 0.9649072 3.10023 0.1037 0.311 0.7594 No

VitD3 -0.02463444 0.01824528 -0.2809 -1.350 0.1947 No

**Analysis of Variance ─────────────────────────────────────────────────────────**

**R² Lost**

**If Term(s) Sum of Mean Prob**

**Source DF Removed Squares Square F-Ratio Level**

Intercept 1 50.41923 50.41923

Model 4 0.4367 5.979433 1.494858 3.295 0.0358

TC_HDL_Ratio 1 0.2525 3.457512 3.457512 7.622 0.0134

BMI 1 0.0108 0.1473199 0.1473199 0.325 0.5762

Waist_Ht_Ratio 1 0.0032 0.04394381 0.04394381 0.097 0.7594

VitD3 1 0.0604 0.8269866 0.8269866 1.823 0.1947

Error 17 0.5633 7.711932 0.453643

Total(Adjusted) 21 13.69136 0.6519697

1. **Sample Size Estimation for the Current Study**

Power Analysis and Sample Size (PASS**)** was used to estimate the sample size

**Multiple Regression using Effect Size**

**Numeric Results ────────────────────────────────────────────────────────────**

Model: Unconditional (Random X's)

**Independent Independent**

**Variables Variables Effect**

**Controlled Tested Size**

**Power N kc kт f² Alpha Beta**

0.8002 762 0 8 0.020 0.050 0.1998

0.8010 111 0 8 0.150 0.050 0.1990

0.8045 54 0 8 0.350 0.050 0.1955

0.9001 966 0 8 0.020 0.050 0.0999

0.9004 139 0 8 0.150 0.050 0.0996

0.9051 67 0 8 0.350 0.050 0.0949

**References**

Gatsonis, C. and Sampson, A.R. 1989. 'Multiple Correlation: Exact Power and Sample Size Calculations.'

Psychological Bulletin, Vol. 106, No. 3, Pages 516-524.

Benton, D. and Krishnamoorthy, K. 2003. 'Computing discrete mistures of continuous distributions: noncentral

chisquare, noncentral t and the distribution of the square of the sample multiple correlation coefficient.'

Computational Statistics & Data Analysis, Vol. 43, Pages 249-267.

Krishnamoorthy, K. and Xia, Y. 2008. 'Sample Size Calculation for Estimating or Testing a Nonzero Squared

Multiple Correlation Coefficient.' Multivariate Behavioral Research, Vol. 43, Pages 382-410.

Cohen, Jacob. 1988. Statistical Power Analysis for the Behavioral Sciences, Lawrence Erlbaum Associates,

Hillsdale, New Jersey.

**Report Definitions**

Power is the probability of rejecting a false null hypothesis.

N is the number of observations on which the multiple regression is computed.

kc is the number of independent variables controlled.

kт is the number of independent variables tested.

Ind. Variables Controlled are those variables whose influence is removed from experimental error.

Ind. Variables Tested are those variables whose regression coefficients are tested against zero.

f² is the effect size. It is calculated using f² = ρ²/(1 - ρ²).

ρ² is squared multiple correlation coefficient of the variables begin tested.

Cohen's f² interpretation: 0.02 = small, 0.15 = medium, 0.35 = large.

Alpha is the probability of rejecting a true null hypothesis. It should be small.

Beta is the probability of accepting a false null hypothesis. It should be small.

Because adj. R2 was 0.3042 in the pilot series, our assumption of intermediate effect size is not unreasonable. As a result, the study required a minimal sample size of 111. An additional number of 11 (10%) was added to account for any exclusions that were possible after enrollment in the study (see below in the section 7 on Subject Enrollment and Study Sites. Hence, the study was planned for a total sample of 122.

1. **Protocol, Ethics Committee and Registration of the Trail**

A protocol for the study was prepared and submitted to the Ethics Committee at Indo-US hospital, Ameerpet, Hyderabad 500016, India in which the first author (S.M.) had additional affiliation. The title of the protocol was “Determinants of Common Carotid Intima-media Thickness (CCIMT) Measured by Ultrasound Echo-tracking Method in Asymptomatic Individuals”. The protocol (protocol No. MHC/CCIMT/001 dated 30 November 2018) was presented to the Ethics Committee on 24^th^ December 2018 and it was approved on 4^th^ January 2019. The study was permitted at two sites: Indo-US hospital (site 1) and Mantha Heart Clinic, 3-4-512/1, Barkatpura, Hyderabad 500027, India (site 2). The study was registered prospectively in a clinical trial registry in India (<http://ctri.nic.in>) maintained by Indian Council of Medical Research. The registry caters to India as well as neighbouring countries that do not have a primary registry of their own. The registry guidelines stipulate that subject enrolment is permitted only after the registration. The registration number was CTRI/2019/02/017420, 4 February 2019 with an expected study duration of one year. Protocol summary may be viewed from a registry using ‘ccimt’ in the key word search <http://ctri.nic.in/Clinicaltrials/advancesearchmain.php>

1. **Subject enrolment and Study Sites:**

A written informed consent was obtained from all the subjects in the language of their choice English or Telugu (local language). Government issued photo identity card containing age or data of birth served as proof for documenting the age. Subjects were essentially volunteers for the study or those who consulted for vascular wellness (preventive cardiology) and were not charged for any services related to the study including laboratory investigations (blood tests, ECG, ultrasound measurement of CCIMT, reporting and counselling. The site 1 is a 150-beded multispecialty hospital and the hospital staff or their close relatives volunteered to participate in the study and were enrolled after screening for eligibility. Mantha Heart Clinic (site 2) is an outpatient cardiology consultation clinic and has a vascular wellness unit. Services at this clinic are provided by the second co-author of this study (SLT) who is a qualified cardiologist and is in practice for the last 23 years. At this site 2, subjects were either volunteers or those who consulted SLT for vascular wellness (preventive cardiology) and were enrolled after screening for eligibility. Although ECGs were obtained immediately after taking the blood sample, they were interpreted in the next 2-3 days. SLT has interpreted all the ECGs and was blinded to the CCIMT findings. Ultrasound imaging for CCIMT of the all the cases was done by the first author (SM) who was unaware of the ECG or laboratory test results as they were not available at the time of imaging. Subsequently, when the laboratory test results were available and vascular wellness report were prepared and the subjects were called again, usually 4-5 days later, for necessary counselling.

The screening commenced on February 5, 2019 and the enrolment ended on December 31, 2020 when the adequate sample was obtained. The following table depicts distribution of the cases at the 2 sites

| **Item** | **Site 1** | **Site 2** |
| --- | --- | --- |
| Period of study | 24 Jun 2019 to 2 Jul 2019  15 Jul 2019 | Rest of the period from 5 Feb 2019 to 31 Dec 2019 excluding those days at site 1. |
| Number of subjects | 52 | 84 |
| Nature of subjects | Volunteers | Volunteers and those who consulted for vascular wellness care |
| Excluded at screening stage | 6 | 8 |
| Excluded at second stage | 1 | 4 |
| Included for final analysis | 45 | 72 |
| CCIMT risk grade 1 | 1 | 0 |
| CCIMT risk grade 2 | 16 | 17 |
| CCIMT risk grade 3 | 20 | 37 |
| CCIMT risk grade 4 | 8 | 18 |

Exclusions that are possible after enrolment can be because of the following reasons:

1. Changes in specific ECG suggestive of ischemia or need for further work-up (non-specific ST-T changes or partial right bundle branch blocks etc. were not considered a reason for exclusion.
2. Serum creatinine ≥2mg/dL
3. Review of past medical records suggestive of treatment for ischemic cardiovascular and neurovascular problems that were not apparent at the time of screening
4. Ultrasound scan time exceeding 30 minutes for both sides, fall in heart rate below 50 beats/min or fall in oxygen saturation by pulse oximetry below 95% and difficulty in capturing the real time image required for auto edge detection. In this study, none of the subjects were excluded because of these criteria related to the scanning.
5. **Initial Multivariable Linear Regression Analysis with 8 Predefined Indicators**

**Multiple Regression Report**

Dependent IMT_Zscore

**Run Summary Report ─────────────────────────────────────────────────────────**

**Item Value Rows Value**

Dependent Variable IMT_Zscore Rows Processed 117

Number Ind. Variables 8 Rows Filtered Out 0

Weight Variable None Rows with X's Missing 0

R² 0.2330 Rows with Weight Missing 0

Adj R² 0.1762 Rows with Y Missing 0

Coefficient of Variation 0.6732 Rows Used in Estimation 117

Mean Square Error 0.7437727 Sum of Weights 117.000

Square Root of MSE 0.8624226

Ave Abs Pct Error 176.444

Completion Status Normal Completion

**Analysis of Variance ─────────────────────────────────────────────────────────**

**R² Lost**

**If Term(s) Sum of Mean Prob Power**

**Source DF Removed Squares Square F-Ratio Level (5%)**

Intercept 1 192.0309 192.0309

Model 8 0.2330 24.40406 3.050507 4.101 0.0003 0.9904

Error 108 0.7670 80.32746 0.7437727

Total(Adjusted) 116 104.7315 0.9028579

**Regression Coefficients T-Tests ──────────────────────────────────────────────────**

**Regression Standard Standard- T-Statistic Reject Power**

**Independent Coefficient Error ized to Test Prob H0 at of Test**

**Variable b(i) Sb(i) Coefficient H0: β(i)=0 Level 5%? at 5%**

Intercept 0.5939775 0.9648333 0.0000 0.616 0.5394 No 0.0936

TC_HDL_Ratio 0.1478523 0.06114098 0.2279 2.418 0.0173 Yes 0.6688

Age 0.01686974 0.01060579 0.1470 1.591 0.1146 No 0.3509

BMI 0.006407091 0.03680618 0.0262 0.174 0.8621 No 0.0534

WHR -1.0806 2.674421 -0.0622 -0.404 0.6870 No 0.0686

VitD3 -0.03003244 0.01303371 -0.2011 -2.304 0.0231 Yes 0.6269

(Smk=1) 0.7014199 0.2587033 0.2479 2.711 0.0078 Yes 0.7664

(DM=1) -0.08259955 0.2525761 -0.0283 -0.327 0.7443 No 0.0621

(gender="Male") 0.1820488 0.1902794 0.0952 0.957 0.3408 No 0.1577

**Regression Coefficients Confidence Intervals ─────────────────────────────────────────**

**Regression Standard Lower 95% Upper 95%**

**Independent Coefficient Error Conf. Limit Conf. Limit**

**Variable b(i) Sb(i) of β(i) of β(i)**

Intercept 0.5939775 0.9648333 -1.318489 2.506444

TC_HDL_Ratio 0.1478523 0.06114098 0.02666033 0.2690444

Age 0.01686974 0.01060579 -0.004152769 0.03789226

BMI 0.006407091 0.03680618 -0.06654914 0.07936332

WHR -1.0806 2.674421 -6.381766 4.220566

VitD3 -0.03003244 0.01303371 -0.05586751 -0.00419736

(Smk=1) 0.7014199 0.2587033 0.1886251 1.214215

(DM=1) -0.08259955 0.2525761 -0.5832491 0.41805

(gender="Male") 0.1820488 0.1902794 -0.1951179 0.5592155

**Normality Tests ─────────────────────────────────────────────────────────────**

**Test Statistic Reject**

**Test to Test Prob H0 at**

**Name H0: Normal Level 20%?**

Shapiro Wilk 0.984 0.1673 Yes

Anderson Darling 0.379 0.4048 No

D'Agostino Skewness 2.056 0.0397 Yes

D'Agostino Kurtosis 0.772 0.4399 No

D'Agostino Omnibus 4.826 0.0896 Yes

**Multicollinearity Report ───────────────────────────────────────────────────────**

**Variance R² Diagonal**

**Independent Inflation Versus of X'X**

**Variable Factor Other I.V.'s Tolerance Inverse**

TC_HDL_Ratio 1.2501 0.2001 0.7999 0.005026023

Age 1.2021 0.1682 0.8318 0.0001512327

BMI 3.1905 0.6866 0.3134 0.001821383

WHR 3.3359 0.7002 0.2998 9.616548

VitD3 1.0723 0.0674 0.9326 0.0002283999

(Smk=1) 1.1767 0.1502 0.8498 0.08998366

(DM=1) 1.0571 0.0540 0.9460 0.08577172

(gender="Male") 1.3938 0.2825 0.7175 0.04867917

1. **Final Multiple Linear Regression Analysis with 3 Selected Indicators**

Dependent IMT_Zscore

**Summary Report ─────────────────────────────────────────────────────────**

**Item Value Rows Value**

Dependent Variable IMT_Zscore Rows Processed 117

Number Ind. Variables 3 Rows Filtered Out 0

Weight Variable None Rows with X's Missing 0

R² 0.2060 Rows with Weight Missing 0

Adj R² 0.1850 Rows with Y Missing 0

Coefficient of Variation 0.6696 Rows Used in Estimation 117

Mean Square Error 0.735856 Sum of Weights 117.000

Square Root of MSE 0.8578205

Ave Abs Pct Error 185.341

**Regression Coefficients T-Tests ──────────────────────────────────────────────────**

**Regression Standard Standard- T-Statistic Reject Power**

**Independent Coefficient Error ized to Test Prob H0 at of Test**

**Variable b(i) Sb(i) Coefficient H0: β(i)=0 Level 5%? at 5%**

Intercept 0.8002995 0.3316273 0.0000 2.413 0.0174 Yes 0.6674

TC_HDL_Ratio 0.1564133 0.05522421 0.2410 2.832 0.0055 Yes 0.8018

VitD3 -0.02627826 0.01252807 -0.1759 -2.098 0.0382 Yes 0.5477

(Smk=1) 0.8407323 0.2407107 0.2971 3.493 0.0007 Yes 0.9336

**Regression Coefficients Confidence Intervals ─────────────────────────────────────────**

**Regression Standard Lower 95% Upper 95%**

**Independent Coefficient Error Conf. Limit Conf. Limit**

**Variable b(i) Sb(i) of β(i) of β(i)**

Intercept 0.8002995 0.3316273 0.143286 1.457313

TC_HDL_Ratio 0.1564133 0.05522421 0.04700415 0.2658224

VitD3 -0.02627826 0.01252807 -0.05109863 -0.00145788

(Smk=1) 0.8407323 0.2407107 0.3638411 1.317624

**Analysis of Variance ─────────────────────────────────────────────────────────**

**R² Lost**

**If Term(s) Sum of Mean Prob Power**

**Source DF Removed Squares Square F-Ratio Level (5%)**

Intercept 1 192.0309 192.0309

Model 3 0.2060 21.57979 7.193262 9.775 0.0000 0.9973

Error 113 0.7940 83.15173 0.735856

Total(Adjusted) 116 104.7315 0.9028579

**Estimated Equation ──────────────────────────────────────────────────────────**

IMT_Zscore =

0.800299525817231 + 0.156413261584106 * TC_HDL_Ratio - 0.0262782565959847 * VitD3 + 0.840732344389344 * (Smk=1)

**Normality Tests ─────────────────────────────────────────────────────────────**

**Test Statistic Reject**

**Test to Test Prob H0 at**

**Name H0: Normal Level 20%?**

Shapiro Wilk 0.982 0.1173 Yes

Anderson Darling 0.331 0.5136 No

D'Agostino Skewness 2.373 0.0177 Yes

D'Agostino Kurtosis 1.495 0.1348 Yes

D'Agostino Omnibus 7.866 0.0196 Yes

**Multicollinearity Report ───────────────────────────────────────────────────────**

**Variance R² Diagonal**

**Independent Inflation Versus of X'X**

**Variable Factor Other I.V.'s Tolerance Inverse**

TC_HDL_Ratio 1.0309 0.0299 0.9701 0.004144442

VitD3 1.0014 0.0014 0.9986 0.0002132926

(Smk=1) 1.0297 0.0288 0.9712 0.07874043

**10**: **Appendix**

Example of CCIMT measurement by echo-tracking and generated data

| Code | Calendar Age (y) | Gender | CCIMT  Right | CCIMT  Left | CCIMT  standard | Z score | Percentile | Interpretation |
| --- | --- | --- | --- | --- | --- | --- | --- | --- |
| 9306570 | 58 | Male | 717* | 531 | 625 | 0.839 | 80 | Increased thickness |


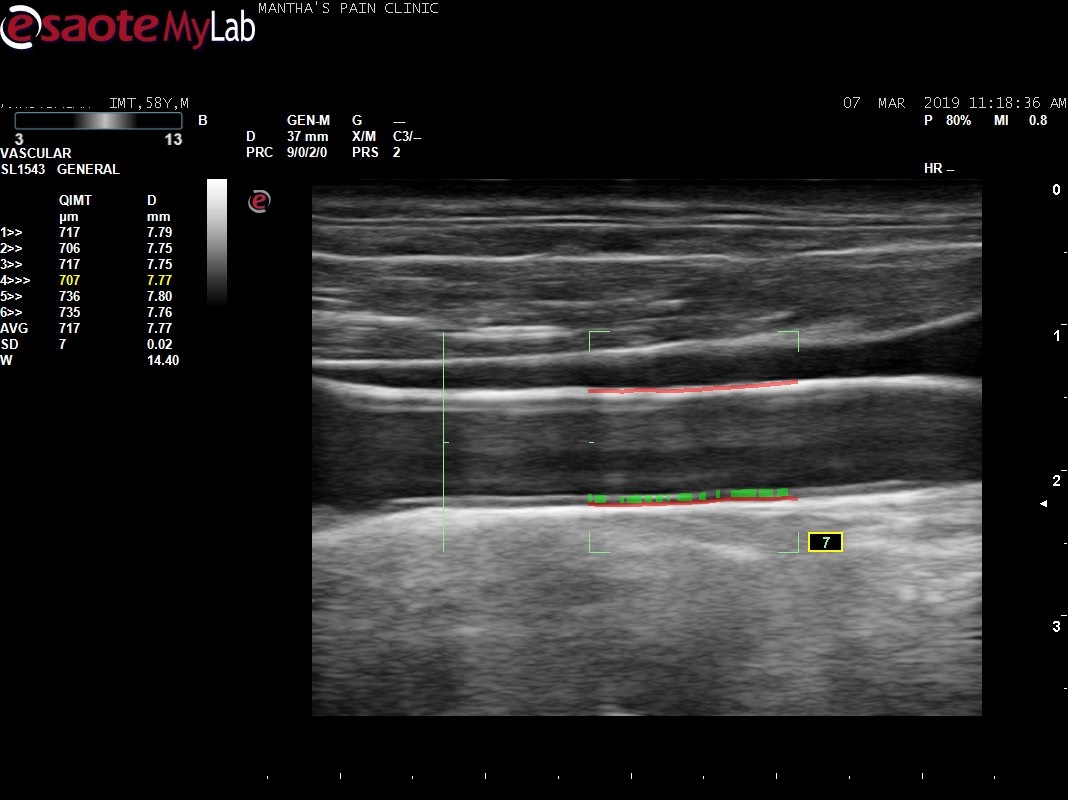


**References:**

1. Darabian S, Hormuz M, Latif MA, Pahlevan S, Budoff MJ. The role of carotid intimal thickness testing and risk prediction in the development of coronary atherosclerosis. *Curr Atheroscler Rep* 2013; **15**(3): 306.

2. Engelen L, Ferreira I, Stehouwer CD, Boutouyrie P, Laurent S, Reference Values for Arterial Measurements C. Reference intervals for common carotid intima-media thickness measured with echotracking: relation with risk factors. *Eur Heart J* 2013; **34**(30): 2368-80.

3. Mantha S, Tripuraneni SL, Fleisher LA, Roizen MF. Use of Common Carotid Intima-Media Thickness Measured by Ultrasound Echo-Tracking in Cardiovascular Risk Stratification Before Noncardiac Surgery in Low-Risk Category: A Research Idea. *A A Pract* 2020; **14**(5): 166-9.

4. Simova I. Intima-media thickness: appropriate evaluation and proper measurement. European Society of Cardiology. 2015. https://www.escardio.org/Journals/E-Journal-of-Cardiology-Practice/Volume-13/Intima-media-thickness-Appropriate-evaluation-and-proper-measurement-described (accessed November 6 2018).

**Digital Content References**

1. Darabian S, Hormuz M, Latif MA, Pahlevan S, Budoff MJ. The role of carotid intimal thickness testing and risk prediction in the development of coronary atherosclerosis. *Curr Atheroscler Rep.* 2013;15(3):306.

2. Engelen L, Ferreira I, Stehouwer CD, Boutouyrie P, Laurent S, Reference Values for Arterial Measurements C. Reference intervals for common carotid intima-media thickness measured with echotracking: relation with risk factors. *Eur Heart J.* 2013;34(30):2368-2380.

3. Mantha S, Tripuraneni SL, Fleisher LA, Roizen MF. Use of Common Carotid Intima-Media Thickness Measured by Ultrasound Echo-Tracking in Cardiovascular Risk Stratification Before Noncardiac Surgery in Low-Risk Category: A Research Idea. *A A Pract.* 2020;14(5):166-169.

4. Simova I. Intima-media thickness: appropriate evaluation and proper measurement. European Society of Cardiology. *e-Journal of Cardiology Practice, Volume 13* 2015; https://www.escardio.org/Journals/E-Journal-of-Cardiology-Practice/Volume-13/Intima-media-thickness-Appropriate-evaluation-and-proper-measurement-described. Accessed November 6, 2018.
